# Early Brain microRNA/mRNA Expression is Region-Specific After Neonatal Hypoxic-Ischemic Injury in a Mouse Model

**DOI:** 10.3389/fgene.2022.841043

**Published:** 2022-02-16

**Authors:** Eric S. Peeples, Namood-e Sahar, William Snyder, Karoly Mirnics

**Affiliations:** ^1^ Department of Pediatrics, University of Nebraska Medical Center, Omaha, NE, United States; ^2^ Department of Pediatrics, Children’s Hospital & Medical Center, Omaha, NE, United States; ^3^ Child Health Research Institute, Omaha, NE, United States; ^4^ Department of Biochemistry and Molecular Biology, University of Nebraska Medical Center, Omaha, NE, United States; ^5^ Department of Pharmacology and Experimental Neuroscience, University of Nebraska Medical Center, Omaha, NE, United States; ^6^ Munroe-Meyer Institute for Genetics and Rehabilitation, University of Nebraska Medical Center, Omaha, NE, United States

**Keywords:** encephalopathy, cerebellum, next-generation sequencing, striatum, cortex

## Abstract

**Background:** MicroRNAs (miRNAs) may be promising therapeutic targets for neonatal hypoxic-ischemic brain injury (HIBI) but targeting miRNA-based therapy will require more precise understanding of endogenous brain miRNA expression.

**Methods:** Postnatal day 9 mouse pups underwent HIBI by unilateral carotid ligation + hypoxia or sham surgery. Next-generation miRNA sequencing and mRNA Neuroinflammation panels were performed on ipsilateral cortex, striatum/thalamus, and cerebellum of each group at 30 min after injury. Targeted canonical pathways were predicted by KEGG analysis.

**Results:** Sixty-one unique miRNAs showed differential expression (DE) in at least one region; nine in more than one region, including miR-410-5p, -1264-3p, 1298-5p, -5,126, and -34b-3p. Forty-four mRNAs showed DE in at least one region; 16 in more than one region. MiRNAs showing DE primarily targeted metabolic pathways, while mRNAs targeted inflammatory and cell death pathways. Minimal miRNA-mRNA interactions were seen at 30 min after HIBI.

**Conclusion:** This study identified miRNAs that deserve future study to assess their potential as therapeutic targets in neonatal HIBI. Additionally, the differences in miRNA expression between regions suggest that future studies assessing brain miRNA expression to guide therapy development should consider evaluating individual brain regions rather than whole brain to ensure the sensitivity needed for the development of targeted therapies.

## Introduction

Neonatal hypoxic-ischemic encephalopathy (HIE), which is the clinical phenotype resulting from perinatal hypoxic-ischemic brain injury (HIBI), is a devastating neurological injury resulting from decreased oxygen and blood flow to the brain around the time of birth. Despite widespread use of therapeutic hypothermia, nearly half of infants with moderate to severe HIE die or suffer from significant developmental disability (Jacobs et al., 2013). Given the continued high morbidity and mortality despite available therapy, novel supplemental therapies are desperately needed. One promising target that has shown potential in *in vivo* studies of HIBI is the modulation of microRNA (miRNA) expression (Li et al., 2019; Xiong et al., 2020).

MiRNAs are small non-coding RNAs that modulate gene expression through post-transcription silencing of messenger RNAs. One significant hurdle to designing miRNA-based therapies for neonatal HIBI is a lack of data describing the endogenous miRNA expression after neonatal HIBI. To date, there have been two studies that have profiled the serum miRNA changes in human newborns with HIE using high-throughput analyses using the same cohort but two different miRNA detection techniques (Looney et al., 2015; Casey et al., 2020). Between the two studies, the investigators identified at least 107 miRNAs that were significantly up- or downregulated in newborns diagnosed with HIE. The human studies, however, are understandably limited to only circulating blood levels and therefore may vary considerably based on the amount of systemic organ (heart, liver, kidney, etc.) injury that results from the hypoxic-ischemic injury (Michniewicz et al., 2020). Our group recently profiled the temporal changes in brain miRNA expression at 24 and 72 h after neonatal HIBI (Peeples et al., 2022); however, no study to date has assessed brain region-specific miRNA changes after injury.

The brain has been shown to have a unique baseline miRNA profile compared to other tissues, and each brain region also has a specific expression signature (Hua et al., 2009). These baseline differences may result in significantly different regional miRNA responses in disease processes, such as neonatal HIBI, that tend to preferentially affect certain regions of the brain more than others. Although all regions of the brain are exposed to the systemic hypoxia that is introduced in the unilateral carotid artery ligation HIBI model, the reduction in blood flow resulting in the ischemic injury does not affect all brain regions equally. In one of the initial descriptions of this model by Dr. Vannucci, his group described the most significant reductions in regional blood flow as occurring in the striatum and thalamus (2–3 times the reduction seen in areas such as the subcortical white matter), which correlated closely with the distribution and extent of ischemic neuronal necrosis that was primarily seen in the striatum, thalamus, and posterior cortical regions (Vannucci et al., 1988). At the same time, Dr. Vannucci’s group noted no change or increased blood flow to the cerebellum and brainstem after HIBI in this model, consistent with an injury produced by occluding only the anterior cerebral circulation (carotid artery). Injury to the thalamus and basal ganglia have been consistently associated with poorer outcomes after HIBI (Martinez-Biarge et al., 2011; Cheong et al., 2012); thus, assessing miRNA expression in different regions may provide further insight into the development of adverse outcomes after HIBI.

Based on this knowledge of the variable blood flow distribution and injury, the goal of the current study was to evaluate the brain region-specific miRNA changes after neonatal HIBI in a mouse model. The primary regions of interest were chosen as the cortex, striatum/thalamus, and cerebellum. We hypothesized that the miRNA profile would differ between the cortex and striatum/thalamus and that the cerebellar miRNA expression would be distinct from both of the other regions given its relative protection from ischemic injury in this model. As described above, all of the human studies were obtained immediately after delivery (i.e., cord blood) (Looney et al., 2015; Casey et al., 2020), but previous studies in the piglet model of neonatal HIBI have demonstrated that many miRNAs do not reach peak dysregulation until around 1 h after injury (Garberg et al., 2017; Casey et al., 2020). As such, to allow for comparison with the previous clinical studies (of cord blood at 0 min after injury) but also ensure time for miRNA dysregulation to occur (peak at 60 min after injury), the region-specific miRNA levels assessed in this study were all obtained at 30 min after injury, consistent with the timing used in other similar studies (Yuan et al., 2010; Garberg et al., 2017).

## Materials and Methods

This study protocol was reviewed and approved by the University of Nebraska Medical Center Institutional Animal Care and Use Committee. Timed pregnant CD1 mouse dams were obtained from Charles River Laboratory (Wilmington, MA). The CD1 strain was chosen due to the high rate of moderate-to-severe injury and low mortality after HIBI (Sheldon et al., 1998). Because the overall goal of this research was to identify potential miRNA targets for therapeutic intervention rather than the identification of biomarkers that can differentiate between hypoxia and HIBI or between levels of HIBI severity, the study was not powered to stratify by severity and a hypoxia-only group was not used. After delivery, pups were maintained in a 12-h light and 12-h dark environment with the dam and littermates. At postnatal day 9, pups of both sexes were randomized to HIBI or control (4 pups/group). The HIBI groups were induced with 5% isoflurane and then were anesthetized with 2.5% isoflurane. Analgesia was provided by injection of 2 mg/kg bupivacaine at the incision site. A small vertical incision was made in the midline of the ventral neck and the neck was dissected in order to identify, isolate, and cauterize the right common carotid artery (Sheldon et al., 1998). Surgeries took no longer than 5 min. The control group underwent the same anesthesia, analgesia, and dissection, but the neck was closed without vessel ligation. Pups were maintained at normothermia (34–36°C skin temperature with infrared thermometry (Goodrich, 1977; Mei et al., 2018)) throughout surgery and recovery. Once the pups were fully recovered, they were returned to the dam and littermates for a 2-h recovery period. After recovery, groups were again separated, and the HIBI group underwent 30 min at 8% oxygen in a hypoxia chamber (BioSpherix, Parish, NY) at normothermia while the sham control group spent the same 30 min separated from the dam but in a warm normoxic environment.

At 30 min after injury, pups were euthanized, the brain extracted quickly and the hemispheres separated. Each hemisphere was further dissected into three regions—cortex, striatum/thalamus, and cerebellum—and then immediately frozen on dry ice and stored at −80°C until analysis. The entire cortex was used for these analyses. The hippocampus was also dissected out but was not able to be analyzed due to low miRNA yield. Regional brain tissue was then homogenized using a dounce homogenizer with Qiazol Lysis Reagent (Qiagen, Hilden, Germany) and the RNA extracted using the RNeasy Lipid Tissue Mini kit (Qiagen) per the manufacturer instructions. The RNA concentration and integrity was determined through spectrophotometry (DeNovix DS-11, Wilmington, DE). Only samples with ratios of 260/280 nm absorbance >1.9 were used for analyses.

The RNA samples then underwent further quality confirmation through parallel capillary electrophoresis on a Fragment Analyzer (Agilent, Santa Clara, CA) and then were processed for high-throughput sequencing. For miRNA-Seq, libraries were prepared from 200 ng RNA per sample with the NEXTFLEX Small RNA Kit (PerkinElmer, Waltham, MA) per the manufacturer’s instructions. The libraries were then sequenced on the Illumina NextSeq 550 platform (San Diego, CA). For the mRNA analyses, RNA samples underwent the CodeSet Hybridization protocol with the nCounter Mouse Neuroinflammation probes (Nanostring, Seattle, WA) overnight and then transferred to the nCounter MAX/FLEX (Nanostring) device for analysis.

mRNA panel findings were validated by qPCR for the mRNAs that were highly differentially expressed and/or were previously reported to have biological relevance in neonatal HIBI. The High-Capacity cDNA Reverse Transcription Kit (Applied Biosystems) was used to generate cDNA from 1 µg of RNA. qPCR was performed using the PowerUp SYBR Green Master Mix (Applied Biosystems, Waltham, MA) with specific primers for each of the target mRNAs (shown in Table 1). The expression of four housekeeping genes (GAPDH, HPRT1, PGK1 and RPLPO) and the efficiency of the primer pairs were examined in samples from HIBI and controls. HPRT1, PGK1 and RPLPO were uniformly expressed between the different experiment conditions but the PGK1 primer pair had the best percent efficiency of 104%, so cycle threshold (Ct) values were normalized to the housekeeping gene PGK1 and expression levels were calculated by the ΔΔCt method (Livak and Schmittgen, 2001). For validation of miRNA-Seq findings, we performed qPCR on five miRNAs: mmu-miR-128-2-5p, mmu-miR-155-5p, mmu-miR-1969, mmu-miR-335-5p, and mmu-miR-6240. We performed qPCR on eight brains per group. miRCURY LNA Reverse Transcription Kit (Qiagen) was used to generate cDNA from 200 ng of RNA. We performed qPCR using the miRCURY LNA SYBR Green qPCR kit (Qiagen) with manufacturer generated primers for each of the target mature miRNAs. Values were normalized to U6 as an endogenous control. Expression fold-change in the HIBI group compared to controls was reported after log_2_ transformation. All qPCR samples were run in triplicate.

**TABLE 1:** Sequences for each messenger RNA (mRNA) quantitative polymerase chain reaction primer.

mRNA	
ATF3 Forward	CCA​GGT​CTC​TGC​CTC​AGA​AG
ATF3 Reverse	CAA​AGG​GTG​TCA​GGT​TAG​CAA
BDNF Forward	ATT​AGC​GAG​TGG​GTC​ACA​GC
BDNF Reverse	TCA​GTT​GGC​CTT​TGG​ATA​CC
EGR1 Forward	GAG​CAC​CTG​ACC​ACA​GAG​TC
EGR1 Reverse	CGA​GTC​GTT​TGG​CTG​GGA​TA
FOS Forward	GGT​GAA​GAC​CGT​GTC​AGG​AG
FOS Reverse	CCT​TCG​GAT​TCT​CCG​TTT​CT
NFKB2 Forward	GGC​CGG​AAG​ACC​TAT​CCT​AC
NFKB2 Reverse	AGG​TGG​GTC​ACT​GTG​TGT​CA
SOCS3 Forward	GGT​CAC​CCA​CAG​CAA​GTT​TC
SOCS3 Reverse	GGT​ACT​CGC​TTT​TGG​AGC​TG
SRXN1 Forward	ATG​TAC​CTG​GGA​GCA​TCC​AC
SRXN1 Reverse	GCT​GCA​TGT​GTC​TTC​TGA​GC

For miRNA sequencing, results were aligned to mature mouse miRNA from miRBase using bowtie2 alignment, and differential expression analysis performed using EdgeR. A false discovery rate <0.05 or *p*-value < .05 was considered significant. Those miRNAs with inverse differential expression in the cortex and striatum/thalamus compared to cerebellum suggested specificity for ischemia, given that the cerebellum undergoes hypoxia but not ischemia in this model. Those with similar direction of differential expression in all three regions were suggested to be primarily affected by the systemic hypoxic injury. For Nanostring mRNA analyses, data were uploaded to nSolver for normalization and analysis. Expression pattern relationships between the three regions were assessed using unsupervised two-way (gene vs. sample) hierarchical clustering based on Euclidian distance using the Morpheus program (Broad Institute, Cambridge, MA). Correlation between Nanostring high-throughput results and qPCR were performed using linear regression with Prism version 9.2.0 (GraphPad, La Jolla, CA). Goodness of fit was determined using the R^2^ value.

The miRNA KEGG pathway analyses were performed using Diana Tools (Vlachos et al., 2015) using Tarbase annotations, a *p*-value threshold of .05, and enrichment analysis by Fisher’s exact test with hypergeometric distribution. The mRNA KEGG pathway analyses were performed using Ingenuity Pathway Analysis (Qiagen). For both analyses, pathway data were stratified by -log (*p*-value). Network visualization of mRNA-mRNA and miRNA-mRNA networks were obtained using Cytoscape (Institute for Systems Biology, Seattle, WA). The mRNAs demonstrating significant differential expression in each region were mapped to the BioGRID *Mus musculus* interactions archive (version 4.4.200). The nodes from the significant mRNAs were selected, and networks were generated using two degrees of connection (undirected) between those mRNA and the other mRNA included in the Nanostring Neuroinflammation panel. Those mRNAs with less than two connections were excluded from the network. Node fill color relates to the log fold change of mRNA in hypoxic-ischemic brain injury compared to controls. The same process was used for the miRNA-mRNA networks, with the following exception: starting with the miRNAs that demonstrated significant differential expression, the miR2Gene pathway analysis (Qiu et al., 2011) was used to predict miRNA-mRNA interactions.

## Results

**FIGURE 1:**
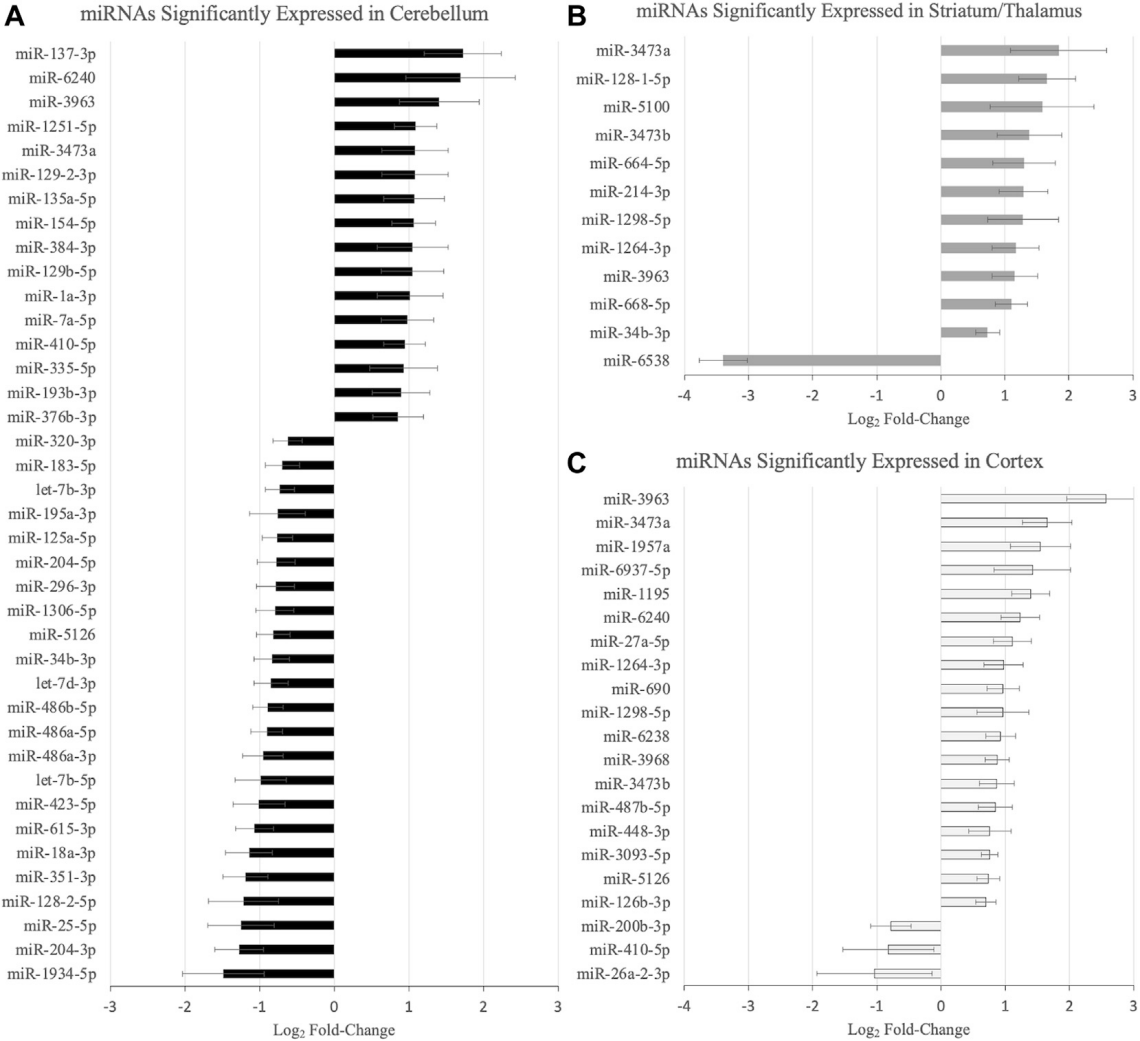
MicroRNAs with significant differential expression at 30 min after hypoxic-ischemic brain injury in the **(A)** cerebellum, **(B)** striatum/thalamus, and **(C)** cortex (*n* = 4/group for each region). Significance defined by fold-change > 1.5, *p* value and/or false discovery rate < .05, and average count per million reads >10. Error bars represent standard error of the mean.

**FIGURE 2:**
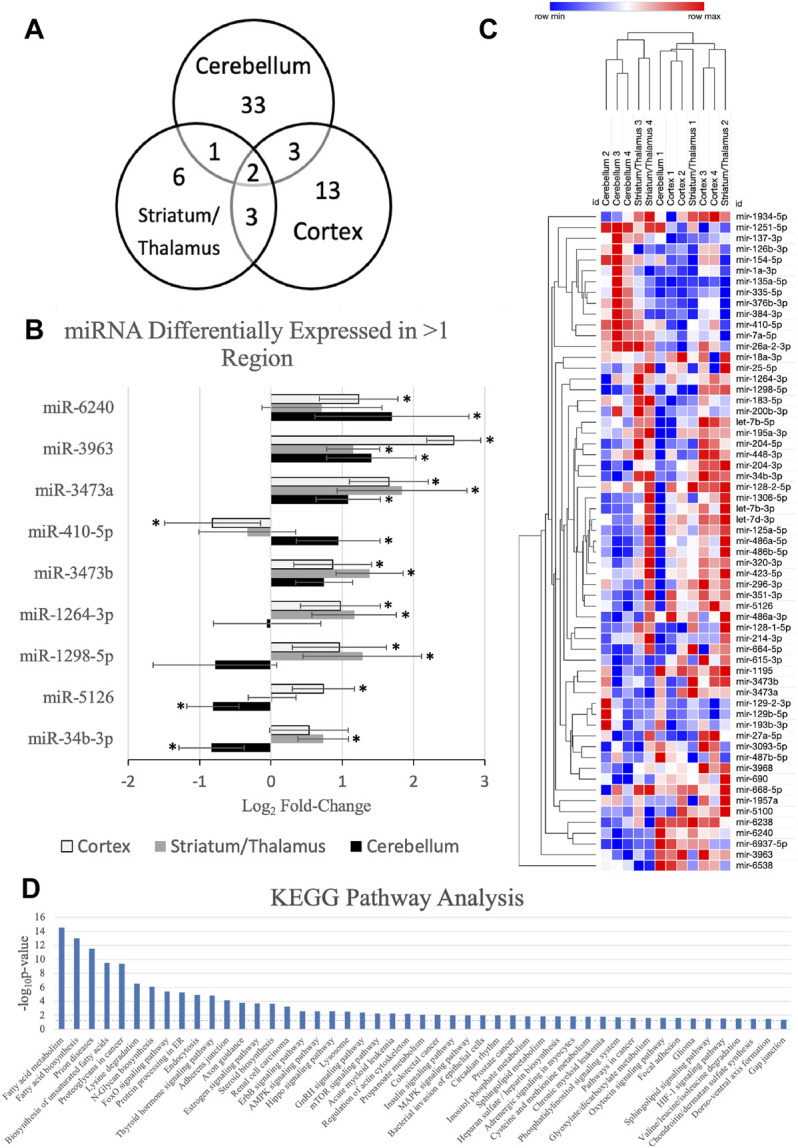
MicroRNAs (miRNA) with significant differential expression in more than one region. **(A)** Venn diagram demonstrating the number of miRNAs with significant differential expression and the overlap between each region; **(B)** differential expression of the nine miRNAs with significant differential expression in more than one region (*regional expression significant, as defined by fold-change > 1.5, *p* value < .05, and average count per million reads >10); **(C)** two-way unsupervised clustering of differentially expressed microRNAs demonstrating separation of the majority of the cerebellum samples from the cortex and striatum/thalamus samples. Rows represent microRNA species and columns represent samples. Each square represents expression in a single sample, color-coded for magnitude of change relative to controls; and **(D)** KEGG pathway analysis of the miRNAs with significant differential expression. Error bars represent standard error of the mean.

Figure 1 demonstrates the miRNAs with significant differential expression after neonatal HIBI compared to controls in each region. There were 39 miRNAs that were differentially expressed in the cerebellum (16 upregulated; 23 downregulated), 12 in the striatum/thalamus (11 upregulated; 1 downregulated), and 21 in the cortex (18 upregulated; 3 downregulated). In all, 61 unique miRNAs demonstrated significant differential expression in one or more region studied (shown in Figure 2A); nine of which had significant differential expression in more than one region. Of those miRNAs affected in multiple regions, miR-410-5p, -1264-3p, -1298-5p, -5,126, and -34b-3p all had inverse differential expression in the cortex and striatum/thalamus compared to the cerebellum. MiR-6240, -3,963, -3473a, and -3473b all had differential expression in the same direction for the three regions (shown in Figure 2B). The differentiation between cerebellar miRNA signaling and that of the cortex and striatum/thalamus was confirmed by two-way unsupervised clustering (shown in Figure 2C) demonstrating a clear separation of three of the four cerebellum samples from those of the other two regions.

Further assessment of the canonical pathways that are predicted to be affected by the miRNAs that were upregulated at 30 min after HIBI was performed by KEGG pathway analysis (shown in Figure 2D). The KEGG analysis demonstrated that the miRNAs with altered regulation in this study primarily alter metabolic pathways, including that of fatty acid metabolism and biosynthesis, lysine metabolism, steroid biosynthesis, AMPK signaling, and sphingolipid metabolism. Many of the traditional hypoxia and cell death pathways, including mTOR, MAPK, and HIF-1 were also included in the KEGG analysis, but had a lower -log (*p*-value).

**FIGURE 3:**
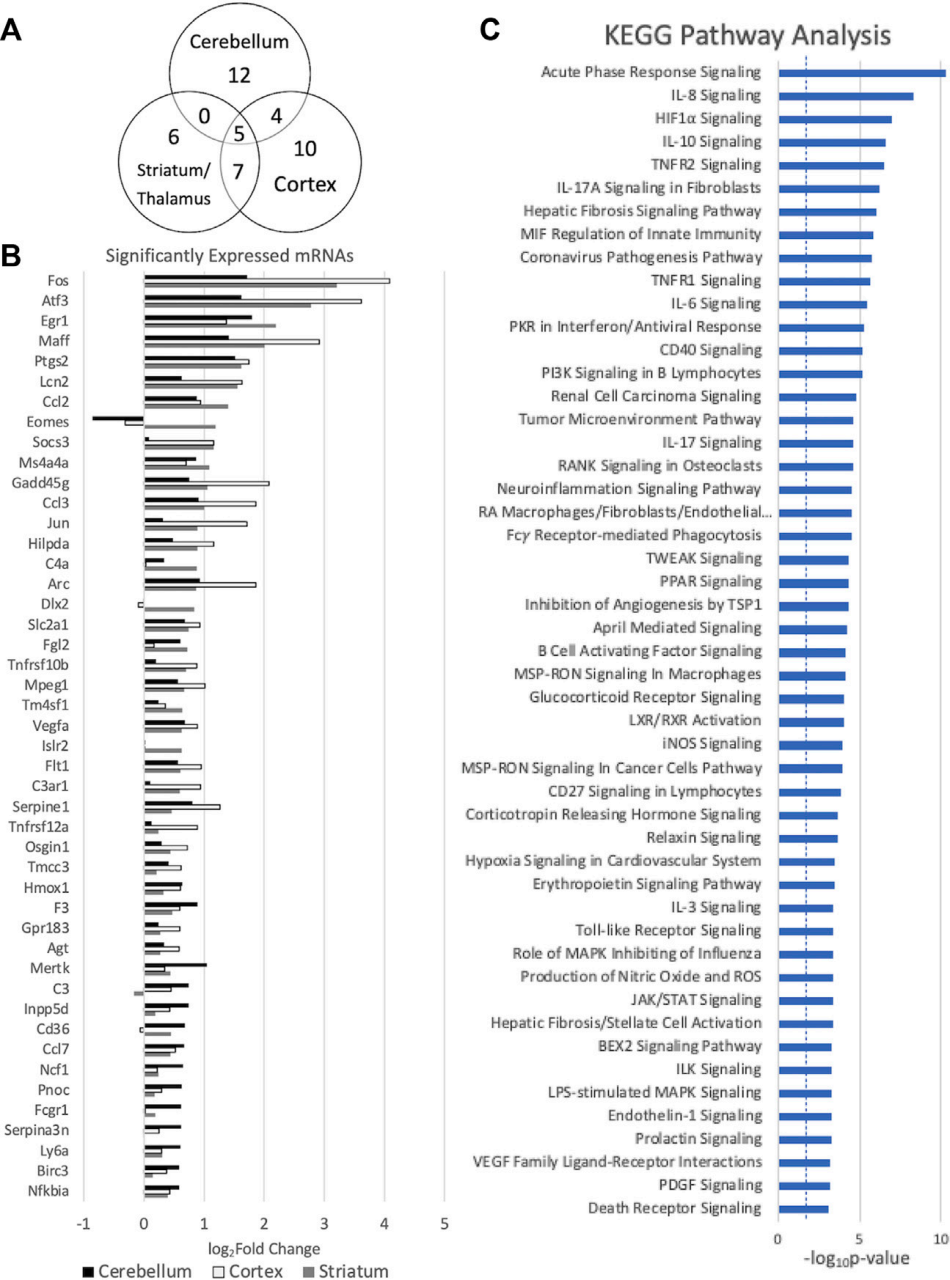
Messenger RNAs (mRNA) with significant differential expression in more than one region. **(A)** Venn diagram demonstrating the number of mRNAs with significant differential expression and the overlap between each region; **(B)** differential expression of the mRNAs with significant differential expression in at least one region (significance defined by fold-change > 1.5 and *p* value and/or false discovery rate < .05), *n* = 4/group for each region; and **(C)** KEGG analysis demonstrating the canonical pathways affected by all of the mRNAs demonstrating significant differential expression. Error bars represent standard error of the mean.

In order to further evaluate the effects of miRNA alterations on mRNA levels in the early period after HIBI, mRNA levels were also assessed at 30 min after injury using the Nanostring Neuroinflammation Panel. Figure 3 shows the 44 mRNAs that were differentially expressed in at least one region; 16 demonstrating significant differential expression in two or more regions (shown in Figure 3A). Of note, all mRNAs that had significant differential expression demonstrated increased expression after HIBI relative to controls (shown in Figure 3B). Only a few mRNAs in the figure showed downregulation in any of the three regions (for instance, Eomes in the cerebellum and cortex); however, in each case this downregulation did not reach statistical significance.

KEGG mRNA pathway analysis demonstrated that the mRNAs that are upregulated at 30 min after HIBI are largely involved in inflammatory and cell death pathways, including interleukin (IL-3, -8, -10, -17A), TNF receptor, HIF1⍺, and PI3K signaling (shown in Figure 3C). For the mRNA KEGG analysis, 335 pathways were identified as significant; the top 50 were included in the figure.

**FIGURE 4:**
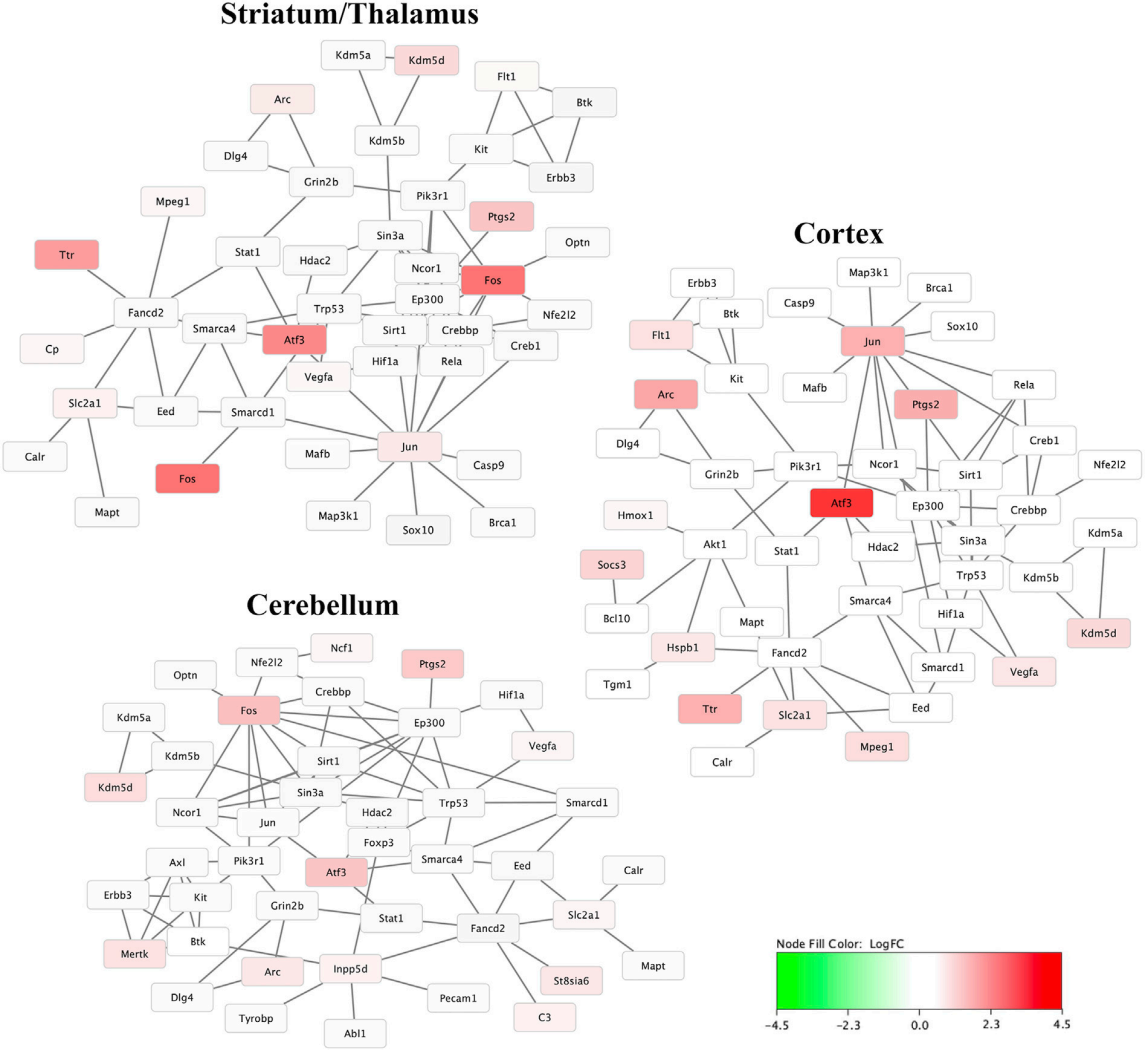
Messenger RNA (mRNA) networks in each region representing the mRNA with significant differential expression after hypoxic-ischemic brain injury versus controls and two degrees of connection between those mRNA and the other mRNA analyzed in this study. Not shown are any mRNAs with no, or only one, connection. Node fill color relates to the log fold change of mRNA in hypoxic-ischemic brain injury compared to controls.

**FIGURE 5:**
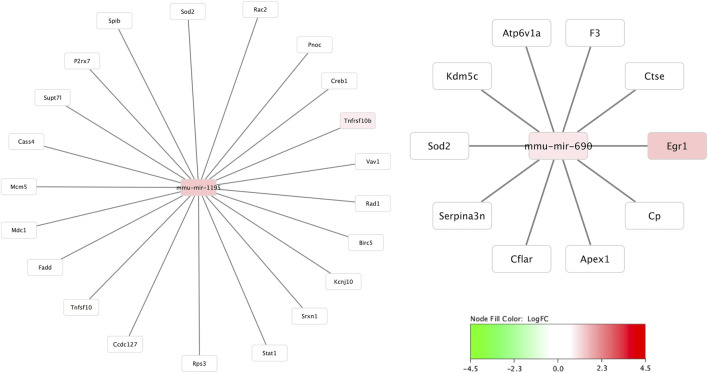
MicroRNA to messenger RNA (miRNA-mRNA) networks in the cortex. The only two miRNAs that demonstrated significant differential expression at 30 min and were closely linked to mRNAs also significantly altered at 30 min after injury were miR-1195 and -690. Neither the striatum/thalamus nor cerebellum expression data resulted in significant miRNA-mRNA networks.

Figure 4 demonstrates the mRNA-mRNA network connections between those mRNAs with significant differential expression 30 min after HIBI and up to two degrees of connection with the other mRNAs analyzed in this study. The differentially regulated genes that were consistently demonstrated to be highly integrated into all three regional networks included Atf3, Fos, Arc, Jun, and Tlr. Two miRNA species that were significantly differentially expressed in the cortex were closely associated with mRNAs that were also found to have significant differential expression: miR-1195 and -690 (shown in Figure 5). MiR-1195 was associated with Tnfsf10b and miR-690 with Egr1. There were no significant miRNA-mRNA networks in either the striatum/thalamus or the cerebellum.

**FIGURE 6:**
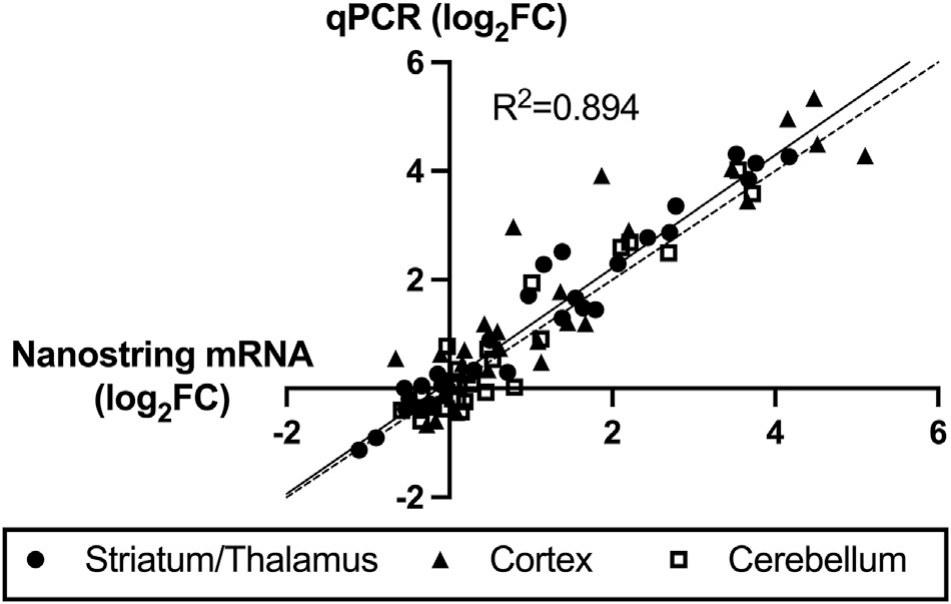
Correlation between Nanostring RNA sequencing and quantitative polymerase chain reaction (qPCR) validation of seven highly expressed mRNAs. The *x*-axis values are Nanostring log_2_ fold-change (FC, hypoxic-ischemic brain injury versus control) values and *y*-axis are qPCR ΔΔCt log_2_FC values. The solid line represents the best-fit regression of the data points with strong correlation (R^2^ = 0.894) and the dashed line represents the ideal correlation line (x = y).

For validation of high-throughput mRNA data, qPCR was performed on each of the samples for seven of the mRNAs with significant differential expression. Figure 6 shows very strong correlation (R^2^ = 0.894) between the Nanostring values and the qPCR values for all three regions and all seven mRNA species. Four miRNAs also demonstrated consistent directional differential expression in striatum/thalamus qPCR compared to miRNA Seq: miR-128-2-5p (log_2_FC -0.36 vs -0.28), miR-155-5p (log_2_FC -0.50 vs. -0.98), miR-1969 (log_2_FC 0.07 vs. 0.44), and miR-335-5p (log_2_FC -0.54 vs. -2.35). One miRNA had inverse direction: miR-6240 (log_2_FC 0.10 in miRNA-Seq vs. -0.98 in qPCR).

## Discussion

This study represents the first brain region-specific profiling of miRNA expression after neonatal HIBI, finding 61 unique miRNAs with significant differential expression in at least one of the three regions studied. Additionally, Nanostring mRNA panel analyses were performed in order to attempt to link the miRNA changes that were seen to alterations in relevant mRNA pathways. Only two miRNAs were found to have direct pathway links to the mRNAs with significant differential expression, underscoring the differences in pathway targeting that were seen between the mRNAs and miRNAs. While the altered mRNAs were mostly made up of immediate early genes (IEG) such as Fos, Jun, and Arc that were associated with downstream inflammatory and apoptotic pathways on KEGG analysis, the miRNAs that demonstrated significant differential expression were mostly associated with pathways of metabolism and synthesis. Although any conclusions regarding pathway analyses must take into consideration the limited scope of the mRNA analyses (using a specific panel rather than whole genome analyses), these data suggest differing roles of mRNAs versus miRNAs in the first 30 min after neonatal hypoxic-ischemic brain injury.

Most of the mRNAs that demonstrated significant differential expression at 30 min after HIBI were IEGs, including Atf3, Fos, Arc, Jun, and Tlr; IEGs that are largely involved in inflammatory and cell death pathways, including interleukin (IL-3, -8, -10, -17A), TNF receptor, HIF1⍺, and PI3K signaling. IEGs are genes that are rapidly transcribed in response to positive (e.g., neuronal activity) or negative (e.g., injury) stimuli and often act as transcription factors and DNA-binding proteins (Minatohara et al., 2016). The regulation of the IEGs is complex but, of importance for the current study, is closely tied with miRNA expression. IEGs can directly regulate the expression of various miRNAs (Avraham et al., 2010). Specifically, Atf3 expression has been shown by multiple investigator groups to be regulated largely by a negative miRNA-based feedback loop (Tindall and Clerk, 2014; Aitken et al., 2015). Stimulating IEG expression briefly inhibits production of the negative feedback miRNAs (Tindall and Clerk, 2014; Aitken et al., 2015). This feedback relationship is a likely cause of the lack of predicted miRNA-mRNA interaction seen in the current study; when IEGs are highly expressed, their associated miRNAs are repressed, and vice versa. Additional studies will be necessary to further investigate the mechanisms for early miRNA- and mRNA-mediated changes (e.g., it is possible that miRNAs do not interact in a typical way with the RNA-induced silencing complex during the acute phase of injury) and to assess miRNA-mRNA interactions at later time points after injury.

Compared to the IEG mRNA expression, the miRNA KEGG analysis demonstrated that the miRNAs with altered regulation were primarily associated with metabolic pathways, which is consistent with the understanding that one of the primary deficiencies in neonatal HIBI is metabolic failure (Sánchez-Illana et al., 2017). The targeted pathways include the ceramide and glycosphingolipid synthetic pathways which are known to be altered by HIBI, with decreased ceramide and sphingomyelin levels present for up to 7 days after preterm HIBI (Vlassaks et al., 2013). The glycosphingolipid pathways are predicted to be altered by miR-6240 (which inhibits Fut1) as well as miR-1264-3p (which inhibits Fut9) (Kanehisa and Goto, 2000). Sterol synthesis and metabolism has also been found to be altered after neonatal HIBI (Dave and Peeples, 2021). One of the primary genes in sterol synthesis is 3-hydroxy-3-methylglutaryl-CoA reductase (HMGCR) may be altered by several of the miRNAs in the current study—including miR-6240, -410-5p, -3473a, -3473b, -1264-3p, and -1298-5p—through the AMPK pathway (Hardie, 2011).

Neonatal HIBI is a triphasic injury. The primary phase starts with the initial hypoxic-ischemic insult leading to primary energy failure and hypoperfusion and continues for the first minutes to few hours after injury. While the secondary phase is primarily characterized by apoptotic cell death, the primary phase consists mainly of necrotic and necroptotic cell death (Hassell et al., 2015). Necroptosis, also termed programmed necrosis, can occur through several pathways and has been shown to be activated by the ErbB (Hu et al., 2021), mTOR (Liu et al., 2014), and HIF1⍺ (Yang et al., 2017) pathways, which were all predicted targets by the differentially expressed miRNAs in our study (shown in Figure 2D). Additionally, miR-214, which demonstrated increased expression in the striatum/thalamus after HIBI in our data has also been associated with necroptosis regulation in other inflammatory diseases (Ou et al., 2019).

To date, there have been very few confirmatory studies of the *in silico* predicted effects of some of the miRNAs with altered expression in multiple regions, including miR-1298, -6240, and -5126. Similarly, although miR-3963 has been found to be highly upregulated in microglia in the developing brain (Varol et al., 2017), it has not otherwise been well studied and it’s conservation between mammalian species is not known. Several of the other promising miRNA targets, however, have demonstrated some potential for neuroprotection in previous studies. For instance, miR-3473a, which was upregulated in all three regions in the current study, was one of the miRNAs found to be downregulated in the brain after preconditioning, suggesting that the relative decrease in expression may play a role in the neuroprotective effects of preconditioning (Miao et al., 2020). Additionally, miR-34b, which demonstrated increased expression in the striatum and thalamus, has been shown to increase cell apoptosis through increased p53 and Bax and decreased BCL2 (Zhang et al., 2020).

Other miRNAs that were differentially expressed in multiple regions include miR-1264, which demonstrated increased expression in the cortex, striatum, and thalamus. Similar to our findings, in adult stroke, upregulated miR-1264 expression was noted immediately after injury, peaking around 3 h after injury (Uhlmann et al., 2017). Inhibition of miR-1264 attenuates apoptosis through targeting WNT/β-catenin signaling (Zou et al., 2021). Lastly, miR-410 was found in the current study to be downregulated in the cortex and upregulated in the cerebellum at 30 min after injury. It was also downregulated in umbilical cord blood (Looney et al., 2015) as well as in circulating blood within 6 h after delivery (Meng et al., 2021) in infants diagnosed with HIE. Overexpression of miR-410 attenuated apoptosis and inflammation in OGD models using both PC12 and SH-SY5Y cell lines (Meng et al., 2021). Furthermore, mesenchymal stem cell derived extracellular vesicles that were neuroprotective when injected intraperitoneally after neonatal HIBI lost their positive effects on cerebral edema and infarct size after treatment with miR-410 antagonist (Han et al., 2021).

When considering the development of miRNA-targeted therapies, it is important to consider timing of intervention. While intervention for neonatal hypoxic-ischemic encephalopathy at 30 min after injury is unlikely to be clinically feasible, the results of the current study can be taken in combination with our previous results (Peeples et al., 2022) to identify those miRNAs that are persistently altered throughout the first few days after injury. For instance, miR-3473b demonstrated increased expression in both the striatum/thalamus and cortex at 30 min after injury and remained elevated at 72 h after injury in our previous study. Consistent with the multiphasic miRNA response that we previously described, however, many of the miRNAs either demonstrated altered regulation early at 30 min without differences at 24 or 72 h (e.g., miR-125a, -410, and -1264) or were altered later at 24 and/or 72 h but did not demonstrate regional differences at 30 min (e.g., miR-182, -2137, and -342). These data further support the importance of taking into account the timing after injury when considering miRNA-based interventions.

In addition to providing insight into potential miRNA targets early after neonatal HIBI, this study also demonstrated that the cerebellum had a unique miRNA profile from either of the other two regions (shown in Figure 2C), likely due to its sparing from the ischemic injury induced by the carotid artery ligation (Vannucci et al., 1988). Our previous study suggested that the contralateral brain is not an appropriate internal control for miRNA studies, at least in the subacute phase after neonatal HIBI (Peeples et al., 2022). Although this study only assessed the miRNA profiles at 30 min after injury, the findings here suggest that contrary the contralateral hemisphere at 24 h after injury, the cerebellum has a distinct miRNA expression profile, at least in the first hour after injury.

There are a few limitations to this study that warrant discussion. Even though the inclusive expression cutoffs (FDR < 0.05 or *p* < .05) in this study minimized type II error in the setting of this exploratory study, this methodology could increase the risk for type I error. Although the goal of the current study was to seek out potential therapeutic miRNA targets for HIBI, the lack of a hypoxia-only control group to allow for better mechanistic understanding could be considered a limitation. Though it may be reasonable to consider the cerebellum group as a hypoxia-only group, in comparing the cortex and striatum/thalamus data to the cerebellum it is not possible to separate whether differences are related to the inter-region differences or due to differences between hypoxia only and HIBI. Additionally, the mRNA analyses in this study were limited to only those mRNAs included in the Nanostring Neuroinflammation panel. Although this panel contains 770 genes relevant to neuroinflammation and brain injury, the network analyses performed were restricted to only those genes in the panel. Also, although all HIBI-injured brains demonstrated visible injury, this study did not include the sample size necessary to stratify results based on characteristics such as severity of brain injury or sex. Though the sample size was limited and therefore may not detect small differences between groups, based on the highly differentially expressed miRNA data (such as miR-2137) from our previous work (Peeples et al., 2022), four animals per group provides 80% power at an alpha level of 0.05 to detect similar differences. Future larger studies would be needed to define whether injury severity or sex affect miRNA expression after HIBI. Lastly, we were unable to study individual hippocampal sections due to low miRNA yield. Future studies assessing hippocampal RNA signaling in this model could consider pooling samples.

In conclusion, this study demonstrated that miRNA expression varies by brain region after neonatal HIBI, and that the cerebellar miRNA profile is clearly distinct from those of the striatum/thalamus and cortex. Although miRNA-mRNA interactions may play more significant roles outside of the immediate period after HIBI, most of the mRNAs with significant differential expression in this study were IEGs associated with downstream inflammatory and cell death pathways that were mostly distinct from the miRNA-targeted metabolic pathways. Future studies assessing brain miRNA expression to guide therapy development should consider evaluating individual brain regions rather than whole brain to ensure the sensitivity needed for the development of targeted therapies.

## Data Availability

The datasets presented in this study can be found in online repositories. The names of the repository/repositories and accession number(s) can be found below: https://www.ncbi.nlm.nih.gov/geo/, GSE184997.
